# Discovering Hidden Diversity of Characins (Teleostei: Characiformes) in Ecuador’s Yasuní National Park

**DOI:** 10.1371/journal.pone.0135569

**Published:** 2015-08-14

**Authors:** Daniel Escobar-Camacho, Ramiro Barriga, Santiago R. Ron

**Affiliations:** 1 Museo de Zoología, Escuela de Biología, Pontificia Universidad Católica del Ecuador, Quito, Pichincha, Ecuador; 2 Museo de Ictiología, Museo de Historia Natural Gustavo Orces, Instituto de Ciencias Biológicas, Escuela Politécnica Nacional, Quito, Pichincha, Ecuador; Australian Museum, AUSTRALIA

## Abstract

**Background:**

Management and conservation of biodiversity requires adequate species inventories. The Yasuní National Park is one of the most diverse regions on Earth and recent studies of terrestrial vertebrates, based on genetic evidence, have shown high levels of cryptic and undescribed diversity. Few genetic studies have been carried out in freshwater fishes from western Amazonia. Thus, in contrast with terrestrial vertebrates, their content of cryptic diversity remains unknown. In this study, we carried out genetic and morphological analyses on characin fishes at Yasuní National Park, in eastern Ecuador. Our goal was to identify cryptic diversity among one of the most speciose fish families in the Amazon region. This is the first time that genetic evidence has been used to assess the species content of the Napo Basin, one of the richest regions in vertebrate diversity.

**Results:**

Phylogenetic analyses of partial mitochondrial 16S ribosomal RNA gene (∼600 pb) DNA sequences from 232 specimens of the family Characidae and its closest groups revealed eight candidate new species among 33 species sampled, representing a 24% increase in species number. Analyses of external morphology allowed us to confirm the species status of six of the candidate species.

**Conclusions:**

Our results show high levels of cryptic diversity in Amazonian characins. If this group is representative of other Amazonian fish, our results would imply that the species richness of the Amazonian ichthyofauna is highly underestimated. Molecular methods are a necessary tool to obtain more realistic inventories of Neotropical freshwater fishes.

## Introduction

Species are the fundamental units in studies of biodiversity, community ecology, and evolutionary biology. An accurate quantification of species diversity is essential for conservation planning since species are used as the operative units of most community or ecosystem level analyses. Efforts to advance the biodiversity inventory are urgent in the Neotropics because they harbor the highest species diversity on Earth [1]. High diversity has been found among a wide variety of taxa including plants, invertebrates, amphibians, mammals, fishes, and birds [1–9]. Completeness of the species inventory is crucial for the management of economically important species and the implementation of conservation measures in a period of global massive extinctions [10,11].

Recent DNA-based studies suggest that tropical faunas contain a large proportion of undescribed species that have escaped detection in morphology based assessments [12–14]. The discovery of this “cryptic diversity” (i.e., separate species that have been classified as a single nominal species because they are, at least superficially, morphologically indistinguishable [15]) suggests that a thorough biodiversity inventory cannot be achieved without modern techniques of species delimitation, specially the use of DNA sequence data. The genetic markers of choice for DNA-based studies are mitochondrial DNA sequences because of their fast rate of evolution [16]. The use of short sequences of DNA for species identification is also known as “DNA Barcoding” [17]. This purely genetic approach has limitations due to the potential incongruences between gene trees and species trees and poor correlation between mtDNA genetic distances and species boundaries [18]. A more reliable approach looks for concordance between genetic characters and other independent sets of characters (e.g., behavioral, morphological). Concordance is indicative of the existence of more than one independent evolutionary lineage because it is unlikely that such a pattern arises by chance. The widespread access to DNA sequence data combined with robust methodologies to define species boundaries is likely to result in a new wave of species discovery comparable to the one that resulted from global exploration by early naturalists during the XVIII and XIX centuries.

Until now, assessments of cryptic diversity in the Neotropical region have focused on terrestrial organisms, especially tetrapods [12,19–23]. In contrast, the content of cryptic diversity in fish faunas, especially in the upper Amazon basin, has not been investigated. Species richness of the Amazonian ichthyofauna is likely underestimated [24,25] and the use of genetic markers to assess cryptic diversity can result in the discovery of a large number of species.

The Neotropical fish fauna has 5,600 formally described species representing more than 50% of the freshwater fishes in the world [6,8]. Within this fauna, the Amazon basin is a hotspot of fish diversity [9] of enormous conservation value. In the western Amazon, the Napo river basin has more than 600 documented fish species [26], 500 of which occur at the Yasuní National Park [27].

Characiformes are a major component of the ichthyofauna in the Amazon Basin. This order, with 2,000 described species [28–30] includes nineteen families [31,32] of which Characidae is the most diverse with 1,100 species. Its alpha taxonomy is dynamic as shown by the description of 250 new species over the last 10 years [29,33–35]. Characidae has taxonomic and phylogenetic problems as a result of its conserved morphology [30,36], which prevents easy species diagnosis. Cryptic diversity has been documented in characiforms. For example, Medrado et al. [37], Ornelas-García et al. [38], Kavalco et al. [39] and Ferreira-Neto et al. [40] working with several species of *Astyanax* encountered abundant cryptic diversity. Pereira et al. [41] also found hidden diversity in *Piabina*. Furthermore, Schneider et al. [42] and Piggot et al. [25] have reported cryptic species in gasteropelecids.

The discovery and inventory of cryptic diversity is likely to have a positive impact in the management of fisheries in the Amazon basin. Accurate identification of a larger number of commercial species should: (i) maximize the number of harvested species, (ii) facilitate the assessment of exploitation rates, and (iii) allow the acquisition of more realistic estimates of sustainable harvesting quotas [43]. These improvements will increase the economic benefits of fish exploitation for Amazonian fishermen [44].

The aim of this study is to assess the content of cryptic diversity in characins of the Amazonian region in Ecuador’s Yasuní National Park. For the first time in this region, characins were analyzed genetically and morphologically to determine its content of cryptic species. The results show a large proportion of cryptic diversity among different lineages of Characidae.

## Materials and Methods

### Identification of Candidate Species

Potential undescribed species were classified using the criteria of Vieites et al. [45] and Padial et al. [46]. A genetic divergence (uncorrected *p* genetic distances for gene 16S) above 3% identified candidate species. Although the Vieites et al. 3% threshold focused on frogs we applied the same value in our study because we sequenced the same gene and found several examples of valid sister species with genetic distances < 3%. Therefore, a 3% threshold is probably conservative and will tend to underestimate the number of candidate species. In addition, previous studies in fish have used 16S with similar approaches [47–49].

We classified candidate species in two categories, confirmed and unconfirmed. Unconfirmed candidate species are those lacking morphologic data that could corroborate the pattern of genetic variation. Confirmed candidate species were those on which we could make morphological comparisons to test their validity as species. Covariation between genetic and morphological data was considered a confirmation of the species status. If covariation was missing we considered them Deep Conspecific Lineages [45]. Morphological analyses were based on specimens deposited at the Ichthyological Museum in Escuela Politécnica Nacional and morphologic and meristic characters were compared with holotype records, see S1 File. We also diagnosed candidate species when putative species were phylogenetically paraphyletic or polyphyletic.

### Study Site, Sample Collection, and Ethics Statement

Permits to carry out this study were obtained from the Ecuadorian Ministry of Environment (document FAUNA No.0030-FAU-MAE-DPO-PNY). This study was evaluated and approved by Pontificia Universidad Católica del Ecuador DGA (Dirección General Académica) in accordance with guidelines for environmental and social impacts for research projects. The Dirección General Académica committee individually evaluates each project to determine its observance of its norms for ethical scientific research. Pontificia Universidad Católica del Ecuador does not have an Institutional Animal Care and Use Committee. Nevertheless, we euthanized all our specimens using lethal dose of buffered MS-222 according to AVMA Guidelines for the Euthanasia of Animals [50] to ensure minimal suffering.

**Fig 1:**
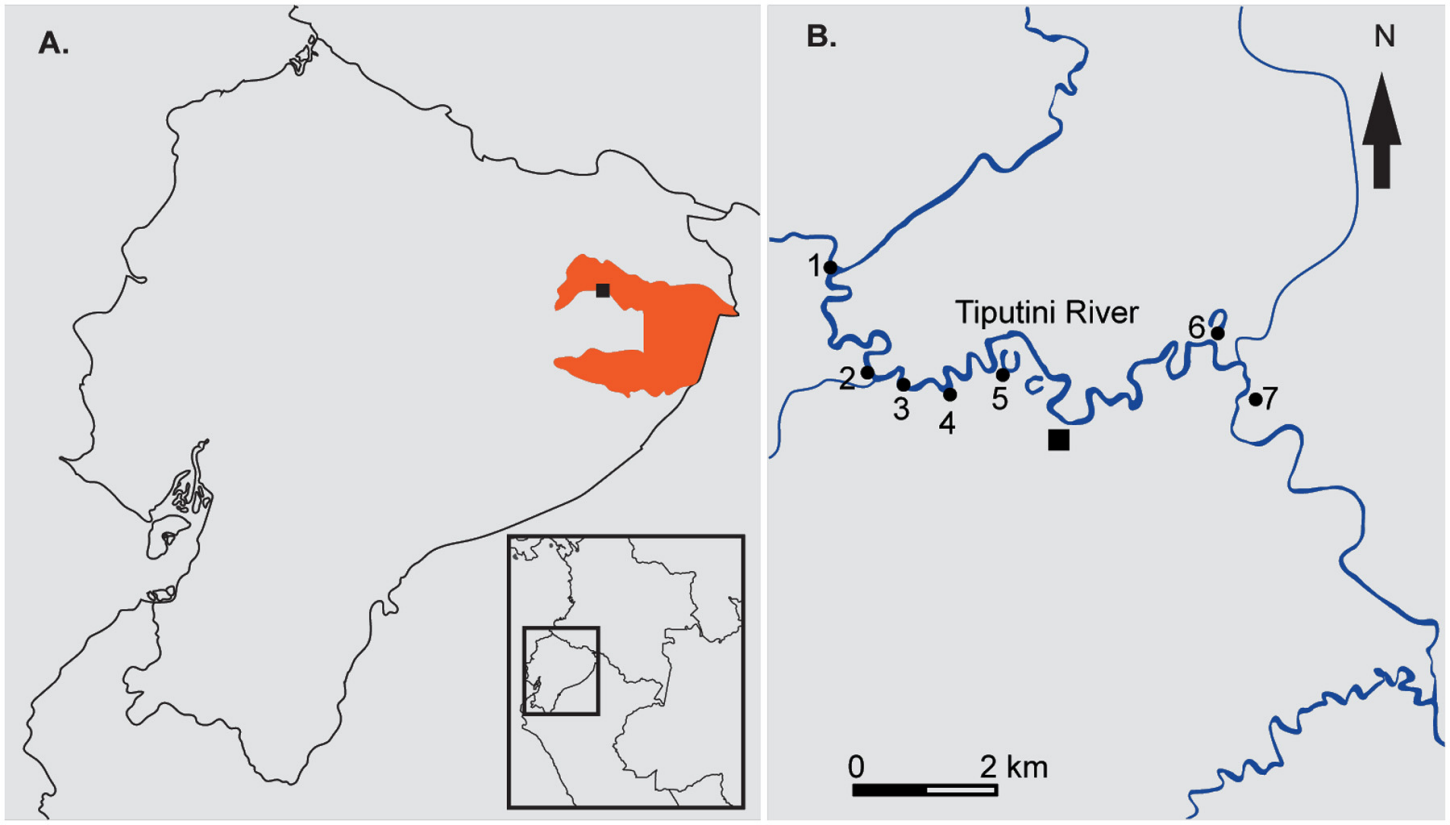
Locality. Map showing the sampled sites at Yasuní National Park. (A) Ecuador showing Yasuní National Park (in orange); (B) the sampling sites in the Tiputini River Basin and Capirón River. Legend: (1) Capirón River, (2–4) Tiputini banks, (5–6) oxbow lakes, (7) black water stream.

We collected 54 specimens of Characiformes from seven sites in the Tiputini River basin, (elevation 220 m above sea level; Fig 1). The Tiputini River is a white water tributary (a river of muddy color with sediment loads from eroding uplands, whereas black water refers to rivers with dark coloration associated with humic acids) of the Napo River in Amazonian Ecuador, in Yasuní National Park. Its meandering course has a highly variable water level [51]. The Napo River is a direct affluent of the Amazon River. We also fished in the Capirón River, two oxbow lakes next to the Tiputini River and one black water stream. Black waters are characterized by low sediment content, acidic pH, and dark coloration from tannins originating in adjacent forests. Fishing techniques included seines, fishing lines, fish traps and manual hand nets. No protected species were collected. For tissue collection, we removed muscle tissue and stored it in 95% ethanol. Upon return from the field, tissues were permanently stored in ultra-freezers at -80°C. Specimens and tissues are deposited in the Museo de Zoología (QCAZ) of Pontificia Universidad Católica del Ecuador in Quito.

### Extraction, PCR, and sequencing

DNA was extracted from muscle tissue using a phenol-chloroform protocol with modifications (M. Fujita, unpublished data). Total DNA was quantified using a Nanodrop ND-1000 (NanoDrop Technologies, Inc.) The mitochondrial gene fragment 16S rRNA was amplified by polymerase chain reaction (PCR). Reactions were performed in a total volume of 25μl using a thermocycler (MyCycler, Thermal Cycler, Bio-Rad). Reactions contained 0.5 μl deoxynucleotide triphosphate (dNTP) (10mM), 2.5 μl PCR buffer 5X (200 mM Tris-HCl (pH 8.4), 500 mM KCl), 0.5μl of each primer (10 μM), 0.25 μl of *Taq* DNA polymerase, 1.5 μl template DNA and 18.25 μl of H_2_O. Conditions were as follows: 94°C (2 min); 35 cycles of 94°C (30 s), 53°C (30 s) and 72°C (1 min), followed by 72°C (7 min). The primers for amplification were 16Sa-L (5′-ACGCCTGTTTATCAAAAACAT-3′) and 16Sb-H (5′-CCGGTCTGAACTCAGATCACGT-3′) [52]. Amplified products were checked on 1% agarose gels stained with SYBR Safe (Invitrogen Life Technologies, Carlsbad, USA). PCR products were purified using the PCR Purification Kit (GE Healthcare; www.gelifesceinces.com) and sequenced at the Macrogen sequencing facility (Macrogen Inc., Seoul, Korea).

### Morphology

Taxonomically useful morphological characters vary with taxa. Hence, we evaluated different morphological characters for each specific group. We took measurements with a digital caliper (to the nearest 0.1 mm) following the methodology of Fink and Weitzman [53]. Greatest body depth and head length in relation to standard length were measured for all groups. Meristic characters included number of branched anal rays, number of lateral line scales, number of teeth in the maxilla, premaxilla, and dentary, and number of scales above and below the lateral line. Lateral-line scale counts include all scales in the series (both pored and non-pored), including scales posterior to the hypural joint. In counting the branched anal-fin rays, the last two rays, which usually meet at the base, were counted as one. We also evaluated caudal spots and dorsal and lateral body color pattern.

### Genetics

We generated sequences for 56 characiform samples. We combined them with 176 sequences from GenBank. We chose as outgroup *Carassius auratus* (Cypriniformes). Sequences were edited in Geneious Pro 5.3.4 (Biomatters Ltd). A preliminary alignment was carried out with MAFFT [54]. The alignment was visually inspected and manually adjusted with Mesquite 2.74 [55]. Uncorrected genetic distances were obtained with Mesquite excluding ambiguous sites.

Phylogenies were inferred using maximum-likelihood (ML) and Bayesian approaches. For both analyses, evolutionary models for one or two partitions (the whole 16S gene, or stems and loops separately) were estimated with jModelTest [56]. Bayes factors were used to choose the best partition scheme. ML analyses were performed in GARLI v.2.0 [57] with 22 replicate analyses. To assess nodal support, non-parametric bootstrap values were calculated with 100 pseudoreplicates.

Bayesian analyses were conducted in MrBayes v.3.2.1 [58] with two replicate searches of 2 x 10^6^ generations each with four Markov chains. Trees were sampled every 1000 generations. We used TRACER, v.1.3 [59] to verify adequate, effective sample sizes for the search (values > 200). We removed 10% of sampled generations as burn-in before summarizing trees.

## Results

The aligned matrix had 232 terminals and 657 bp of length. The percentage of invariable sites was 46.8%. Out of 349 variable sites, 281 were parsimony informative. The average percentage of missing data was 13% (range 8 to 34).

**Fig 2:**
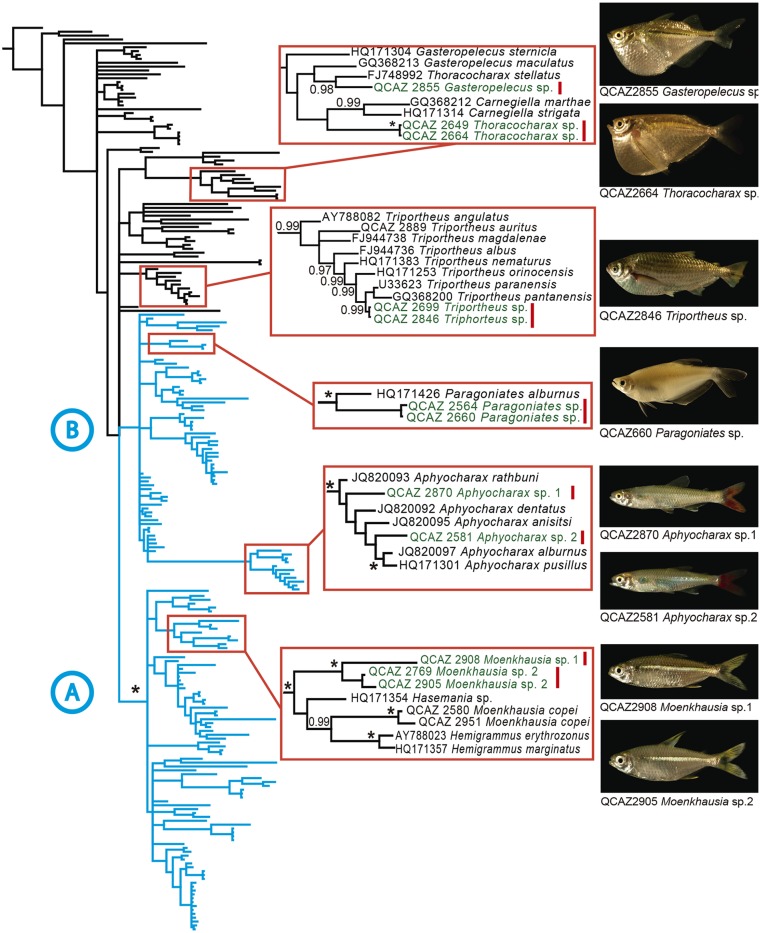
Phylogeny showing uncovered cryptic species. Bayesian consensus phylogram of 231 characins based on DNA sequences of the mitochondrial gene 16S. Red squares show the clades where candidate species were found. Candidate species (inset photos) are shown in green with a vertical red bar.

We discovered eight candidate species (S1 File) among 33 described species, representing an increase in species richness of 24%. No deep conspecific lineages were found. The candidate species belong to the genera *Moenkhausia*, *Aphyocharax*, *Paragoniates* (Characidae), *Thoracocharax* (Gasteropelecidae), *Triportheus* (Triportheidae), and *Gasteropelecus* (Gasteropelecidae) (Fig 2).

The range of uncorrected genetic distances between the candidate species and their closest relatives was 3% to 11%. Morphological examination of the candidate species allowed us to identify six of them as confirmed candidate species. Two confirmed candidate species belong to the *Moenkhausia dichroura* group. Both have genetic distances in excess of 8% relative to their closest relatives, *Moenkhausia lepidura* and *Moenkhausia dichroura*. The new *Moenkhausia* differ from other members of the group and between themselves in color pattern of the caudal lobes, body depth, and number of scales in the lateral line. We also found two new species of *Aphyocharax*. *Aphyocharax* sp. 1 (QCAZ 2870) and *Aphyocharax* sp. 2 (QCAZ 2581) are morphologically similar to *Aphyocharax pusillus* and *Aphyocharax alburnus* respectively. *Aphyocharax* sp.1 has a 4% genetic distance relative to *Aphyocharax pusillus* while *Aphyocharax* sp. 2 has a 3% relative to *Aphyocharax alburnus*. For details on morphological differences see S1 File.

*Paragoniates* has been regarded as a monotypic genus. Our specimens QCAZ 2564 and 2660 show a genetic distance of 5.8% relative to *Paragoniates alburnus*. They also differ in the number of anal branched rays and lateral scales so we consider them to represent a candidate new species. A new species of hatchet fish, *Thoracocharax* sp. (Gasteropelecidae) was also uncovered. The genetic distance between the Yasuní sample and its closest relative, *Thoracocharax stellatus* from Brazil, is 8%. *Thoracocharax* sp. differs in the color of the dorsal and caudal fins and body depth.

The unconfirmed candidate species were *Triportheus* sp. (Triportheidae) and *Gasteropelecus* sp. (Gasteropelecidae). Both showed high genetic distances (3.9% and 7.0%, respectively) relative to other populations of their putative species: *Triportheus albus* and *Gasteropelecus sternicla*. Morphology was inconclusive to determine their taxonomic status. Thus, these species are considered unconfirmed until more data are available.

## Discussion

### Cryptic Diversity in the Amazon Basin

Among the few genetic studies of cryptic diversity in Amazonian fishes, ours is unique in geographic and taxonomic scope and has the highest number of uncovered candidate species (Table 1). By analyzing genetic and morphological data (S1 File) we show that our confirmed candidate species are indeed new and not simply deep conspecific lineages or named species that have not yet been sequenced. With one exception (García-Dávila et al. 2013), all previous analysis of genetic barcoding in Amazonian fishes have been carried out in Central Amazonia (Table 1). Our study is the first targeting a highly speciose clade in western Amazonia, a large geographic region with the highest species diversity of vertebrates at a global scale [27,60].

**Table 1:** Genetic studies of cryptic diversity in Amazonian fish.

Taxa	Percentage increase	Genetic Marker	Region	Reference
(*Pseudoplatystoma* sp.) Pimelodidae	100% (1D/1U)	COI, CR, 7 MS loci	Western Amazon (Iquitos region)	Garcia-Dávila et al. 2013
Characiformes	24% (25D/8U)	16S	Western Amazon (Yasuní)	This study
(*Paratrygon aiereba*) Potamotrygonidae	200% (1D/2U)	ATPase 6, COI	Central Amazon Solimoes, Negro, Tapajos, Xingu, and Araguaia River	Frederico et al. 2012
(*Centromochlus megalops*) Auchenipteridae	500% (1D/5U)	ATPase 6 and 8, RAG 1	Central Amazon: Amazon, Negro and Madeira River	Cooke et al. 2012
(*Pachyurus squamosissimus*) Scieaenidae	100% (1D/1U)	ATPase 6 and 8, RAG 1	Central Amazon: Amazon and Negro River	Cooke et al. 2012
(*Carnegiella marthae*) Gasteropelecidae	200% (1D/2U)	4 MS loci, ATPase 6 & 8	Central Amazon: Negro River	Piggot et al. 2011
(*Carnegiella strigata*) Gasteropelecidae	100% (1D/1U)	ATPase 6 & 8	Central Amazon: Negro River	Schneider et al. 2012

The “Percentage Increase” column shows the number of described (D) species (above bar) and the number of undescribed (U) species (below bar).

Cryptic diversity seems to be widespread among Amazonian fish. It has already been documented in Gymnotiformes [61–63], Siluriformes [64–66], Perciformes [67], and Characiformes [25,42,68,69]. The increase in species richness is frequently as high as 200% (Table 1). If these findings are representative of Amazonian fish in general, we could expect an increase of ~520 to 4300 species in the entire basin (assuming a 24% to 200% increase over its currently described species richness, 2173 species [70]). Such large numbers demonstrate the need for extensive genetic surveys to achieve a more complete inventory of Amazonian fish.

Our study yielded the lowest increase of cryptic taxa among similar studies in the Amazon basin. We suspect that our results underestimate the number of cryptic species for two reasons. First, our geographic sampling was limited. At Yasuní we sampled seven sites over a small area with limited representation of habitat types. Given that Amazonian fish tend to be habitat specialists [6], an increase of localities sampled would lead to an increase in number of species, including undescribed taxa. Second, our study differed from others in targeting an entire order instead of genera or species complexes. As a result, our sampling was less selective at the species level. A more exhaustive and targeted sampling could increase the proportion of cryptic species found. An additional factor that could influence the proportion of cryptic species is water turbidity. Morphologically cryptic species are particularly frequent when chemical communication is more developed than visual communication [15]. Speciation then usually favors divergent selection on mate recognition signals among closely related species. When mate recognition is primarily driven by chemical signals, divergent selection should not influence visual signals and therefore closely related species should display cryptic morphology [15]. Therefore, the white water floodplains forests in the Amazon, which are characterized by turbid water conditions, should have a higher content of cryptic diversity than ecosystems with more transparent water.

In this study, specimens were assigned to particular genera using morphological diagnostic characters. However, in our phylogeny *Moenkhausia*, *Gasteropelecus*, and *Thoracocharax* are non-monophyletic (S1 Fig). Lack of monophyly has been denoted before for *Moenkhausia* [30,35,36,71] and gasteropelecids [72]. Hence, a taxonomic review for each group is needed including samples from the western Amazon region.

We used 16S rRNA as a genetic marker because it has been widely used for identification and discrimination of fish species [16,73–75]. We applied a genetic distance threshold of 3% to identify candidate species. Our threshold is likely very conservative (e.g., prone to underestimate the number of candidate species) because barcode analyses in fish have shown that distances over 2% for the COI mitochondrial gene separate distinct species in 99% of the comparisons [76]. The 16S gene evolves at a slower rate than COI [77]. Therefore, a divergence of 3% in 16S should correspond to a higher divergence in COI, well above the 2% threshold that characterize separate species in Neotropical fish [78].

### The conservation value of Yasuní National Park

Yasuní National Park has an outstanding global conservation importance. Among other records, Yasuní has the highest local diversity for trees, bats amphibians and reptiles [27]. A single hectare of forest could have over 100,000 species of insects (T. Erwin, pers. comm.). Moreover, current estimates of species richness do not consider cryptic diversity. Our results and those of other studies, e.g. [13], suggest a significant underestimation of species richness. Thus, the conservation value of Yasuní could be even higher than currently acknowledged.

Unfortunately, this diversity is threatened by human activities. Oil fields have been exploited in western Yasuní for more than 20 years and new oil fields are being opened in the more pristine areas of the park, the ITT Block, at Yasuní’s eastern region [79]. Such new developments create serious conservation threats for Yasuni’s biodiversity. The need for expanding studies on fish communities in the upper Amazon basin becomes more urgent under these new threats.

## Supporting Information

S1 Fig Phylogeny of Characiformes. Ecuadorian samples are shown in green, GenBank samples in black. Numbers at the branches are clade posterior probabilities (asterisks for 1.0 values).
(PDF)

S1 File Morphological comparisons for confirmed candidate species. (PDF)

S1 Table GenBank accession numbers. (PDF)
