# Supplementary figures and images for: Discovering Hidden Diversity of Characins (Teleostei: Characiformes) in Ecuador’s Yasuní National Park
Source: PLoS One. 2015 Aug 14;10(8):e0135569. doi: 10.1371/journal.pone.0135569 (PMC4537159; doi:10.1371/journal.pone.0135569)

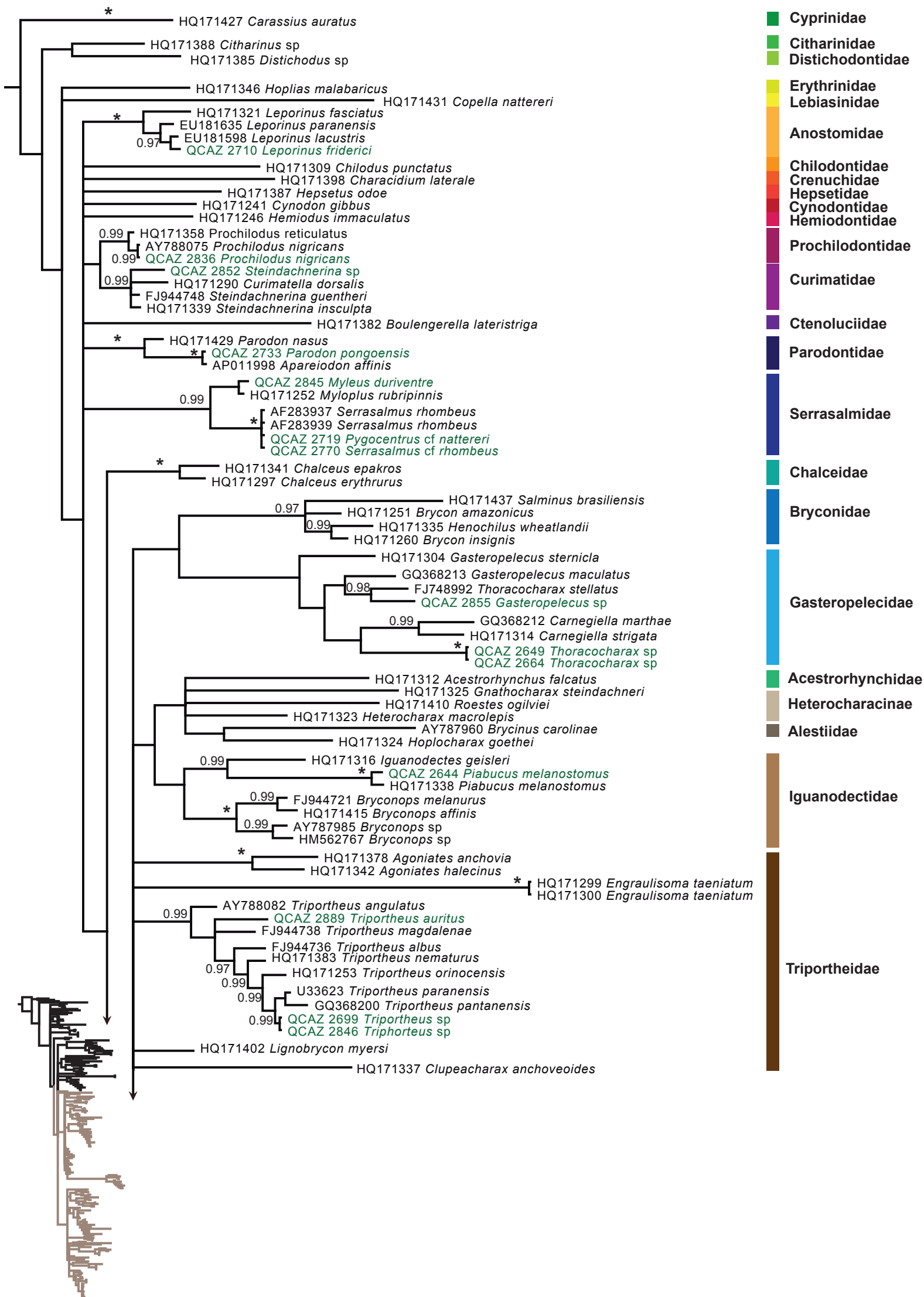

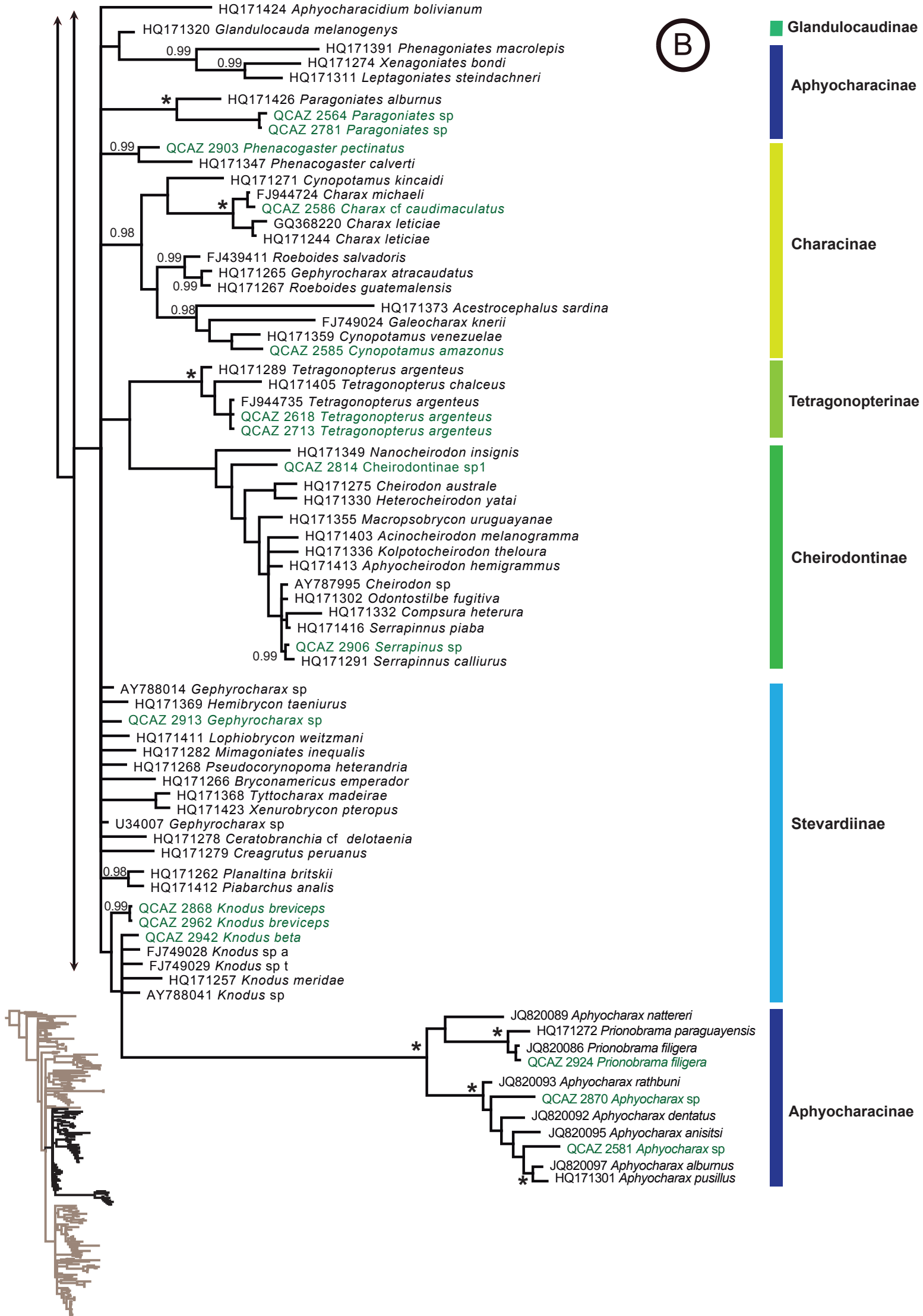

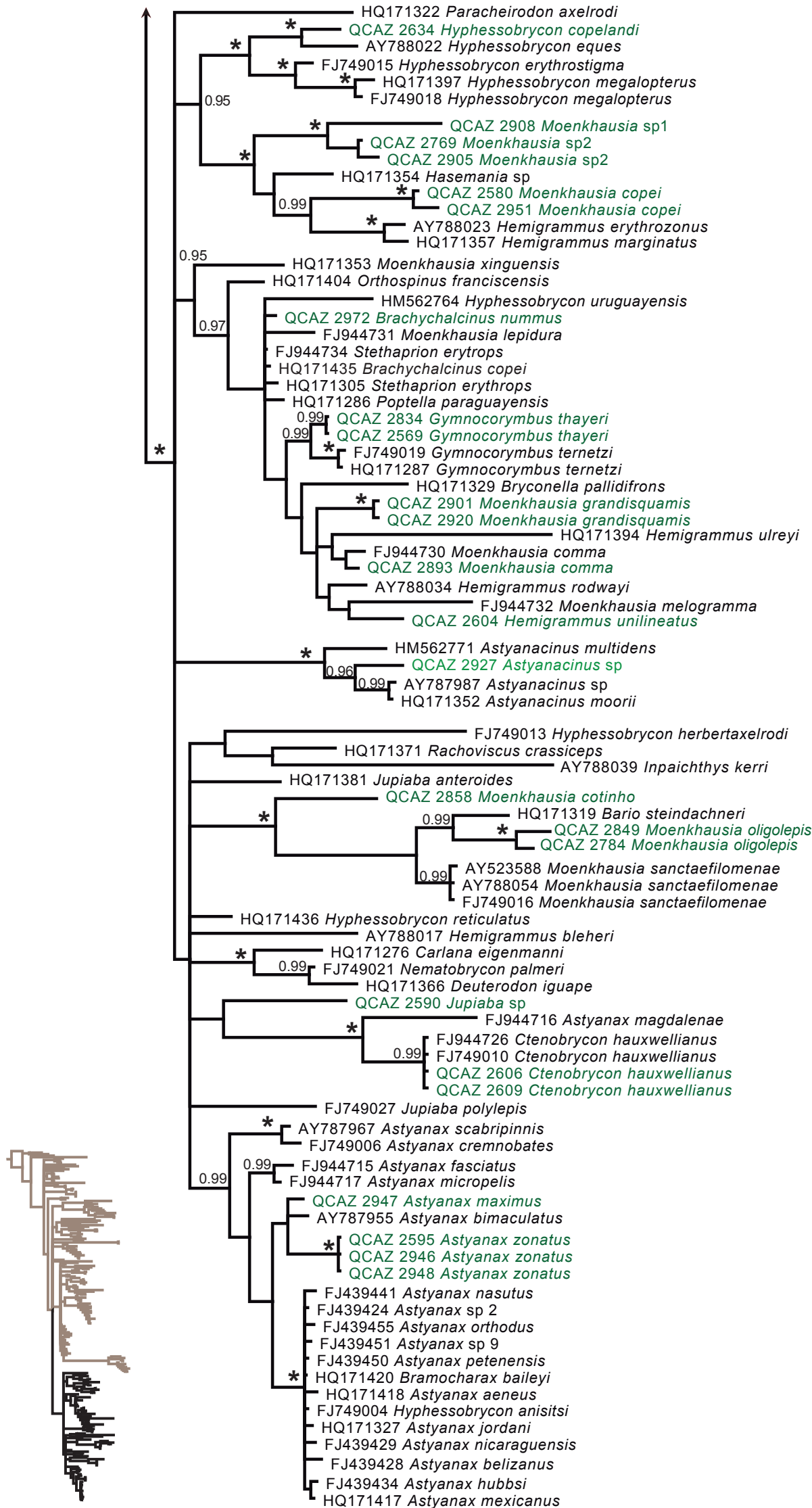

A

Stethaprioninae

Supplement: S1 Fig — Ecuadorian samples are shown in green, GenBank samples in black. Numbers at the branches are clade posterior probabilities (asterisks for 1.0 values). (PDF) [file pone.0135569.s001.pdf]
