# Supplementary material for: Discovering Hidden Diversity of Characins (Teleostei: Characiformes) in Ecuador’s Yasuní National Park
Source: PLoS One. 2015 Aug 14;10(8):e0135569. doi: 10.1371/journal.pone.0135569 (PMC4537159; doi:10.1371/journal.pone.0135569)
Supplement: S1 File — (PDF) [file pone.0135569.s002.pdf]

Supporting Information

File S1.

Variables	Species	
	Aphyocharax sp. n = 10	*Aphyocharax pusillus*
Pored lateral line scales	11 (11-13)	8
Lateral line scales	38.2 (37-39)	36
Maxillary Teeth	13.6 (11-15)	5-8
	Aphyocharax sp. 2 n = 10	*Aphyocharax alburnus*
Dentary teeth	12.1 (10-14)	16-20
Lateral line scales	35.3 (34-36)	37-40
	Paragoniates sp n = 18	*Paragoniates alburnus*
Anal-fin rays	44.4 (42-47)	50
	Thoracocharax sp n = 13	*Thoracocharax stellatus*
Dorsal-fin rays	14.46 (14-15)	13-14
Anal-fin rays	41.46 (38-44)	38-40
Eye diameter in head	3.32 (3.1-3.5)	4
	Moenkhausia sp. 1 n = 26	*Moenkhausia dichroura*
Depth in total length	4.2 (3.9-4.5)	3.3
Head length in total length	5.2 (4.9 – 5.8)	5.5-6
Lateral line scales	32.84 (31-36)	36-38
	Moenkhausia sp. 2 n = 5	*Moenkhausia dichroura*
Lateral line scale	33 (32-34)	36-38
Head in total length	5.22 (4.9-5.3)	5.5-6

Differences in meristic and morphological variables between candidates and previously reported species in the Amazon basin.

***Aphyocharax* sp. 1.** This species is most similar to *Aphyocharax pusillus* by the presence of a black caudal blotch in the middle caudal rays which extends towards

the end of their tips. Rest of the caudal fin is red in life. *Aphyocharax* sp1 differentiates further from *A*. *pusillus* by the presence of a dark blotch on the supraoccipital process, in the number of lateral lines scales, number of pored lateral line scales and in the number of maxillary teeth. *Aphyocharax* sp1 also has more than half of the maxillary toothed while *A*. *pusillus* has one third or two fifths of the maxilla toothed [1,2]. Aphyocharax sp1 differs from *Aphyocharax alburnus* in the coloration pattern having the latter a complete red caudal-fin. *Aphyocharax* sp1 differentiates from *Aphyocharax dentatus*, *Aphyocharax agassizi*, *Aphyocharax nattereri* and *Aphyocharax paraguayensis* by the position of the tip of the maxilla which in *Aphyocharax* sp1, does not reach the second suborbital bone. By the number of teeth in the maxilla *Aphyocharax* sp1 differs from *Aphyocharax rathbuni*, *Aphyocharax rubripinnis*, and *Aphyocharax avary* [1-3].

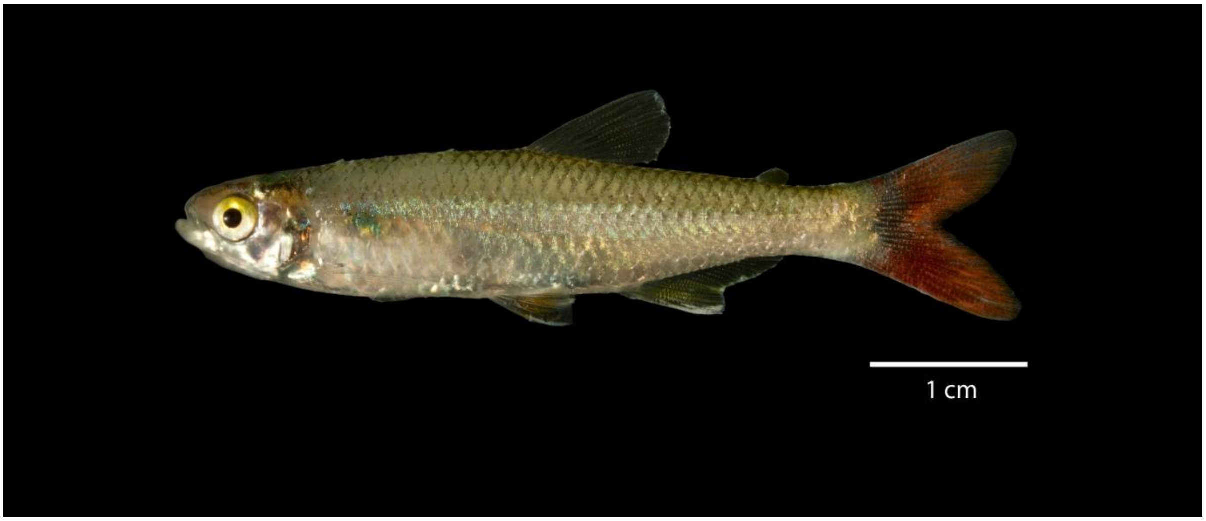

***Aphyocharax* sp. 2.** This species can be distinguished to all its congeners except *Aphyocharax alburnus* by the combination of characters: red caudal-fin in life, tip of the maxilla not reaching the second suborbital bone, maxilla not fully toothed with more than four maxillary teeth. The number of teeth in the dentary and number of lateral line scales can distinguish *Aphyocharax* sp2 from *Aphyocharax alburnus*. Furthermore, the humeral spot on *Aphyocharax* sp2 is faint or absent in contrast with the well-developed humeral spot of *Aphyocharax alburnus* [1,2].

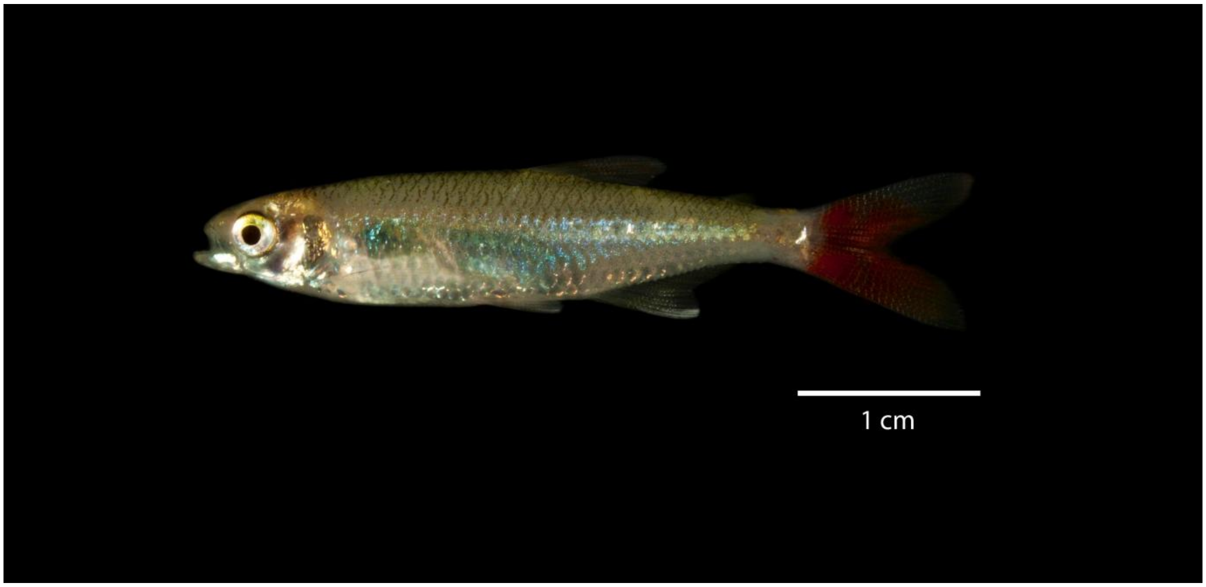

***Paragoniates* sp.** *Paragoniates* is considered a monotypic genus with the only species *Paragoniates alburnus*. *Paragoniates* sp can be distinguished from the former species by the number of anal rays. *Paragoniates* sp lacks of pored lateral line scales, in contrast with 12-13 pored lateral line scales of *Paragoniates alburnus* [2,4,5]. Furthermore, *Paragoniates* sp. exhibits a dusky coloration pattern in the dorsal-fin and the caudal-fin, specially the lower lobe.

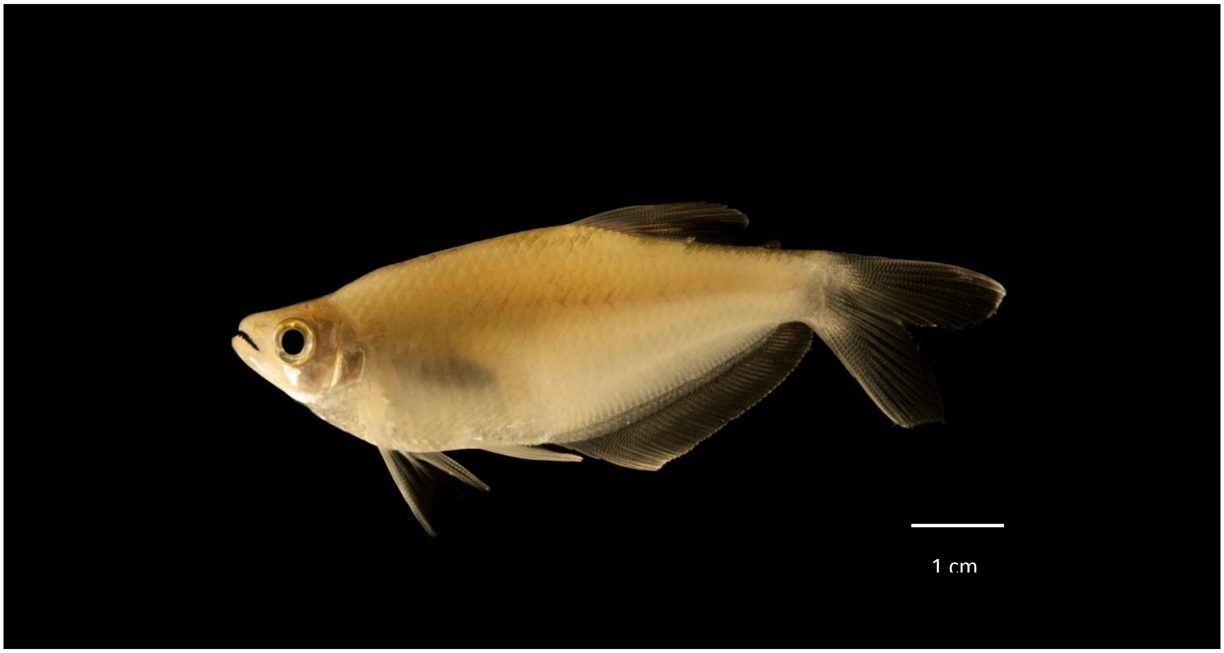

***Thoracocharax* sp.** *Thoracocharax* sp can be distinguished from *Thoracocharax securis* by the black spot in the dorsal-fin, where the first rays of the dorsal-fin are black, and are extended fading at the end of the dorsal-fin whereas in *Thoracocharax* securis the black spot is at the end of the dorsal-fin and not at the base [2,6,7]. *Thoracocharax* sp is also distinguished by the presence of two to three scales covering the anal-fin base vs four to five in *Thoracocharax securis*. *Thoracocharax* sp has the eye diameter 3.3 times in head length vs *Thoracocharax stellatus* with 4. Besides this character, *Thoracocharax* sp has only slight morphological differences with *Thoracocharax stellatus*, varying in the number of dorsal and anal-fin rays. For corroborating data, 12 extra variables were measured and compared with a morphometric study done by Da Silva et al. 2009, where it compares measurements across five different populations of *Thoracocharax stellatus* [7]. Our measurements were also different to the variables in the respective study. *Thoracocharax* sp is furthermore distinguished from the only two species for the genera, *Thoracocharax securis* and *Thoracocharax stellatus* by the dusky caudal lobes, pectoral-fins and snout.

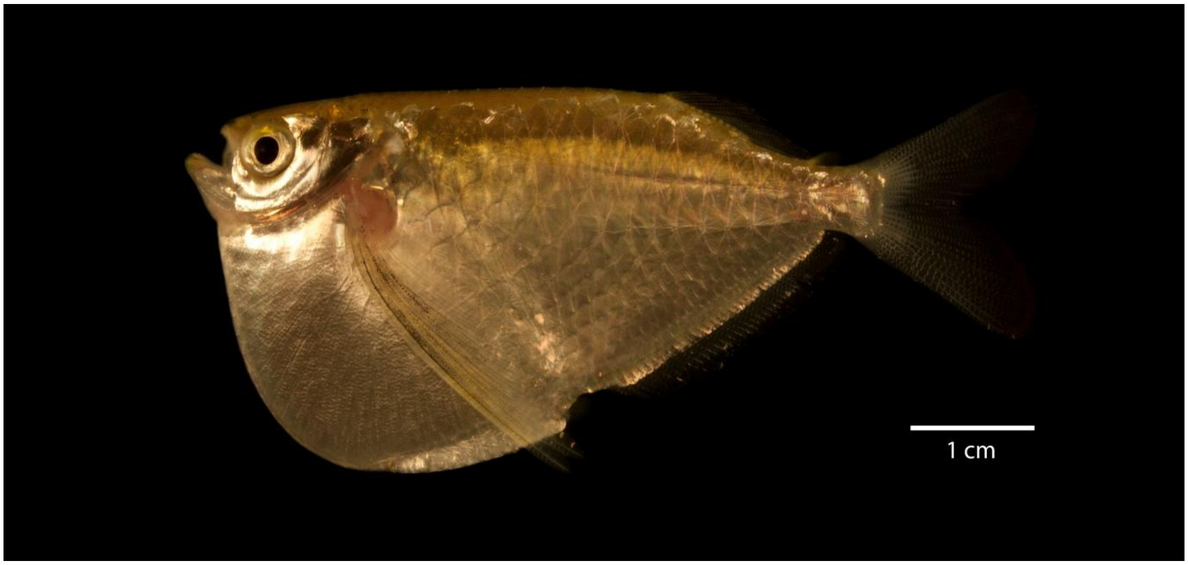

***Moenkhausia* sp. 1:** *Moenkhausia* sp1 belongs to *Moenkhausia lepidura*-group which are small species with body depth usually more than 2.75 in the standard length. *Moenkhausia* sp. 1 is distinguished from all congeners in *Moenkhausia*

lepidura-group, except *Moenkhausia collettii*, *Moenkhausia dichroura* and *Moenkhausia intermedia* by a slender body (3-3.5 times in SL), 31-36 lateral line scales, five scales above and three scales below the lateral line; and a silver midlateral band. *Moenkhausia* sp. 1 differs from *Moenkhausia collettii* in head 3.7-4.5 times in SL (vs. 3.5-3.8), one or two teeth on maxilla (vs. 2-3), and 22-26 anal rays (vs. 24-34) [2,8-11]. *Moenkhausia* sp. 1 further differs from *Moenkhausia intermedia* and *Moenkhausia dichroura* by its dusky-grey color of the caudal fin in contrast with the black marked caudal lobes of the previously mentioned species.

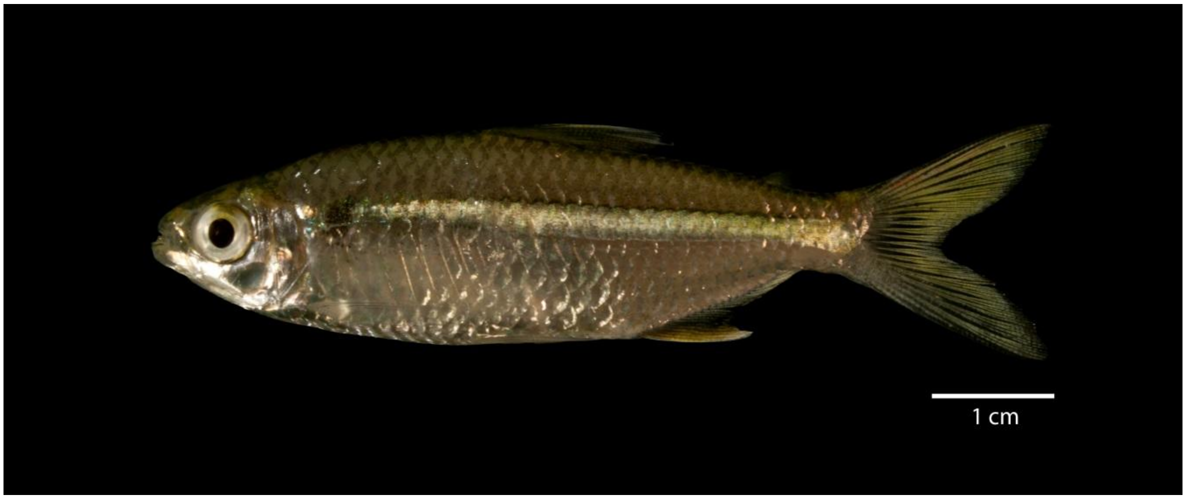

***Moenkhausia* sp. 2.** *Moenkhausia* sp. 2 belongs to *Moenkhausia lepidura*-group. *Moenkhausia* sp. 2 differs from all congeners in *Moenkhausia lepidura*-group, except *Moenkhausia dichroura*, *Moenkhausia intermedia* and *Moenkhausia bonita* by its black marked caudal lobes, depth 2.6-2.7 times in SL, 24-26 anal rays, humeral spot and a silver lateral line. *Moenkhausia* sp. 2 can be distinguished from *Moenkhausia dichroura* by the number of lateral lines scales (32-34 vs 36-38) and head, 4.9-5.3 times (vs 5.5-6) in total length [2,9-11]. *Moenkhausia* sp. 2 differs from *Moenkhausia intermedia* by the tip of the maxilla which does not reach beyond the anterior margin of the eye; and from *Moenkhausia bonita* by its coloration pattern. *Moenkhausia* sp. 2 show a silver midlateral band, a light body pigmentation and a humeral spot [2,9-11]. Additionally, *Moenkhausia* sp. 2 exhibits a dusky-grey blotch at the middle caudal rays which distinguishes from other congenerics. *Moenkhausia* sp. 1 and Moenkhausia sp. 2 inhabit the Tiputini River,

Napo Basin, with *Moenkhausia cotinho*, *Moenkhausia oligolepis*, Moenkhausia comma and Moenkhausia chrysargyrea [12], all of which are deep bodied species that differ from *Moenkhausia* sp. 1 and *Moenkhausia* sp. 2 in numerous details. *Moenkhausia cotinho* can be easily differentiated by the incomplete lateral line and the black caudal spot.

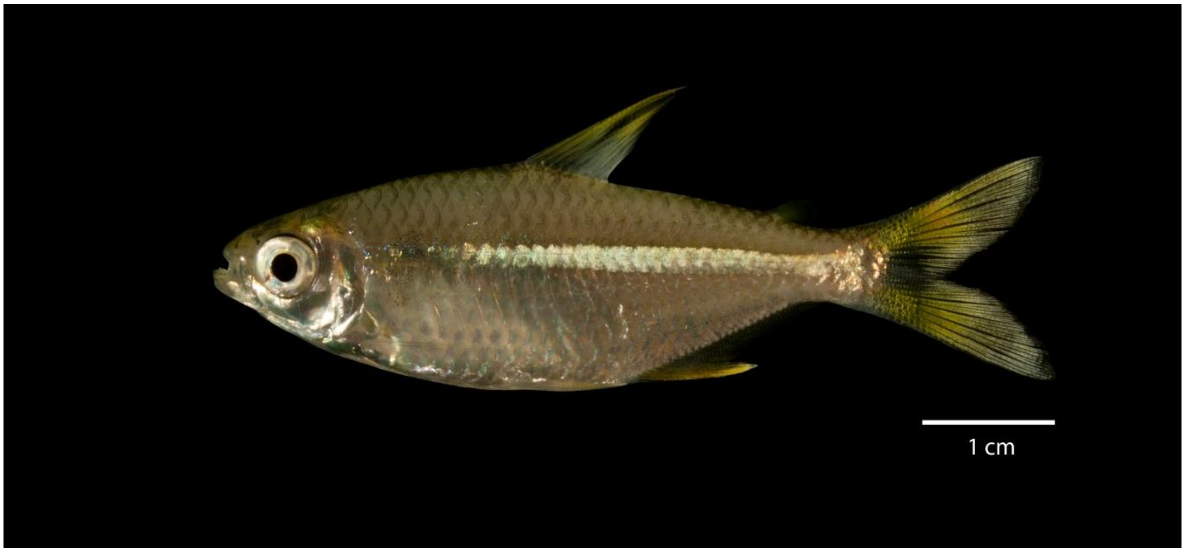

Supplementary References:

1. Günther A. Report on a collection of Fishes made at St. Helena by J.C. Meliss. Proc. Zool. Soc. Lond. 1868: 225-247.
2. Géry J. Characoids of the world. 1st ed. Neptune City: T.F.H. Publications. 1977.
3. Tagliacollo VA, Lima-Souza R, Benine RC, Oliveira C. Molecular phylogeny of Aphyocharacinae (Characiformes: Characidae) with morphological diagnoses for the subfamily and recognized genera. Mol. Phylogenetic. Evol. 2012;64: 297-307.
4. Steindachner F. Ichthyologische Beiträge (V). Sitzb. Akad. Wiss. Wien. 1876;74: 49-240.
5. Eigenmann CH. The Cheirodontinae, A Subfamily of Minute Characid Fishes of South America. Memoirs of the Carnegie Museum 1915;7: 99.

6. Fowler, HW. Further Knowledge of some heterognathous fishes. Part II. Proc. Acad. Nat. Sci. Philadelphia. 1907;58: 431-483.
7. Da Silva EL, Centofante L, Miyazawa CS. Análise morfométrica em *Thoracocharax stellatus* (Kner, 1858) (Characiformes, Gasteropelecidae) proveniente de diferentes bacias hidrográficas Sul-americanas. Biota Neotrop, 2009;9: 71-76.
8. Kner R. Beiträge zur Familie der Characinen. Sitzungsber. Akad. Wiss. Wien. 1958;30: 75-80.
9. Kner R. Zur Familie der Characinen. Sitzungsber. Akad. Wiss. Wien. 1859;32: 163-168.
10. Géry J. Description de deux nouvelles espèces proches de *Moenkhausia lepidura* (Kner) (Poissons, Characiformes, Tetragonopterinae), avec une revue du groupe. Revue fr. Aquariol. 1992;19: 69-78.
11. Eigenmann, CH. Zoological results of the Thayer Brazilian Expedition, Preliminary descriptions of new genera and species of tetragonopterid characins. Bull. Mus. Comp. Zool. 1908;52: 91-106.
12. Barriga R. Lista de peces de agua dulce e intermareales del Ecuador. Rev Politecnica. 2012;30: 83-119.
