# Supplementary material for: Discovering Hidden Diversity of Characins (Teleostei: Characiformes) in Ecuador’s Yasuní National Park
Source: PLoS One. 2015 Aug 14;10(8):e0135569. doi: 10.1371/journal.pone.0135569 (PMC4537159; doi:10.1371/journal.pone.0135569)
Supplement: S1 Table — (PDF) [file pone.0135569.s003.pdf]

Table S1. **Sequences used Voucher information and GenBank accession numbers for 232 fish species used in this study.**

Voucher Number	Specimen	Accession Number	Reference
LBP6876-33172	*Acestrocephalus sardina*	HQ171373	Oliveira *et al*., 2011
LBP4191-23707	*Acestrorhynchus falcatus*	HQ171312	Oliveira *et al*., 2011
LBP8104-37551	*Acinocheirodon melanogramma*	HQ171403	Oliveira *et al*., 2011
LBP550326594	*Agoniates halecinus*	HQ171342	Oliveira *et al*., 2011
LBP6740-33471	*Agoniates anchovia*	HQ171378	Oliveira *et al*., 2011
--	*Apareiodon affinis*	AP011998	Nakatani *et al*., 2011*
LBP9055-42219	*Aphyocharacidium bolivianum*	HQ171424	Oliveira *et al*., 2011
LBP830640025	*Aphyocheirodon hemigrammus*	HQ171413	Oliveira *et al*., 2011
LBP-15819	*Aphyocharax alburnus*	JQ820097	Tagliacollo *et al*., 2012
LBP-25524	*Aphyocharax anisitsi*	JQ820095	Tagliacollo *et al*., 2012
LBP-3604	*Aphyocharax dentatus*	JQ820092	Tagliacollo *et al*., 2012
LBP-22132	*Aphyocharax nattereri*	JQ820089	Tagliacollo *et al*., 2012
LBP4046-22920	*Aphyocharax pusillus*	HQ171301	Oliveira *et al*., 2011
LBP-36496	*Aphyocharax rathbuni*	JQ820093	Tagliacollo *et al*., 2012*
QCAZ2870	*Aphyocharax* sp1	KT277920	Present Study
QCAZ2581	*Aphyocharax* sp2	KT277919	Present Study
LBP5783-28195	*Astyanacinus moorii*	HQ171352	Oliveira *et al*., 2011
MUSM-35742A	*Astyanacinus multidens*	HM562771	Thomaz *et al*., 2010*
AMNH233243	*Astyanacinus* sp	AY787987	Calcagnotto *et al*., 2005
QCAZ2927	*Astyanacinus* sp1	KT277932	Present Study
LBP8938-42019	*Astyanax aeneus*	HQ171418	Oliveira *et al*., 2011
Amot10211	*Astyanax belizanus*	FJ439428	Ornelas-García *et al.,* 2008

--	*Astyanax bimaculatus*	AY787955	Calcagnotto *et al*., 2005
MCP-41307	*Astyanax cremnobates*	FJ749006	Javonillo *et al*., 2010
--	*Astyanax fasciatus*	FJ944715	Vera *et al.,* 2009*
A706	*Astyanax hubbsi*	FJ439434	Ornelas-García *et al.,* 2008
LBP451124599	*Astyanax jordani*	HQ171327	Oliveira *et al*., 2011
--	*Astyanax magdalenae*	FJ944716	Vera *et al.,* 2009*
QCAZ2947	*Astyanax maximus*	KT277928	Present Study
LBP8937-42016	*Astyanax mexicanus*	HQ171417	Oliveira *et al*., 2011
--	*Astyanax micropelis*	FJ944717	Vera *et al.,* 2009*
Aman175	*Astyanax nasutus*	FJ439441	Ornelas-García *et al.,* 2008
Aaman55vi	*Astyanax nicaraguensis*	FJ439429	Ornelas-García *et al.,* 2008
JJ28	*Astyanax orthodus*	FJ439455	Ornelas-García *et al.,* 2008
523GU	*Astyanax petenensis*	FJ439450	Ornelas-García *et al.,* 2008
--	*Astyanax scabripinnis*	AY787967	Calcagnotto *et al*., 2005
QCAZ2595	*Astyanax zonatus*	KT277927	Present Study
QCAZ2946	*Astyanax zonatus*	KT277925	Present Study
QCAZ2948	*Astyanax zonatus*	KT277926	Present Study
CPOG-2009-Amaq2339	*Astyanax* sp 2	FJ439424	Ornelas-García *et al.,* 2008
911GU	*Astyanax* sp 9	FJ439451	Ornelas-García *et al.,* 2008
LBP4389-24187	*Bario steindachneri*	HQ171319	Oliveira *et al*., 2011
LBP7094-34623	*Boulengerella lateristriga*	HQ171382	Oliveira *et al*., 2011
LBP192-8853	*Brachychalcinus copei*	HQ171435	Oliveira *et al*., 2011
QCAZ2972	*Brachychalcinus nummus*	KT277890	Present Study
LBP8940-42025	*Bramocharax baileyi*	HQ171420	Oliveira *et al*., 2011
RUSI-065125	*Brycinus carolinae*	AY787960	Calcagnotto *et al*., 2005
LBP2187-15565	*Brycon amazonicus*	HQ171251	Oliveira *et al*., 2011

LBP2369-16075	*Brycon insignis*	HQ171260	Oliveira *et al*., 2011
LBP275418528	*Bryconamericus emperador*	HQ171266	Oliveira *et al*., 2011
LBP4646-24696	*Bryconella pallidifrons*	HQ171329	Oliveira *et al*., 2011
LBP262-4168	*Bryconops affinis*	HQ171415	Oliveira *et al*., 2011
--	*Bryconops melanurus*	FJ944721	Vera *et al.,* 2009*
DC-2004	*Bryconops* sp	AY787985	Calcagnotto *et al*., 2005
ATT-2010-MCP-35087-595	*Bryconops* sp	HM562767	Thomaz *et al*., 2010*
LBP9215-43160	*Carassius auratus*	HQ171427	Oliveira *et al*., 2011
LBP3300-19864	*Carlana eigenmanni*	HQ171276	Oliveira *et al*., 2011
LBP 4199 23601	*Carnegiella marthae*	GQ368212	Abe *et al.,* 2009*
LBP4200-23798	*Carnegiella strigata*	HQ171314	Oliveira *et al*., 2011
LBP3257-20042	*Ceratobranchia* cf *delotaenia*	HQ171278	Oliveira *et al*., 2011
LBP5443-26504	*Chalceus epakros*	HQ171341	Oliveira *et al*., 2011
LBP4211-22727	*Chalceus erythrurus*	HQ171297	Oliveira *et al*., 2011
LBP7614-36938	*Characidium laterale*	HQ171398	Oliveira *et al*., 2011
QCAZ2586	*Charax* cf *caudimaculatus*	KT277902	Present Study
LBP-1480-12700	*Charax leticiae*	GQ368220	Abe *et al.,* 2009*
LBP1480-12700	*Charax leticiae*	HQ171244	Oliveira *et al*., 2011
--	*Charax michaeli*	FJ944724	Vera *et al.,* 2009*
LBP3115-19803	*Cheirodon austral*	HQ171275	Oliveira *et al*., 2011
AMNH233214	*Cheirodon* sp	AY787995	Calcagnotto *et al*., 2005
QCAZ2814	*Cheirodontinae* sp 1	KT277922	Present Study
LBP4090-23527	*Chilodus punctatus*	HQ171309	Oliveira *et al*., 2011
LBP7528-35374	*Citharinus* sp	HQ289289	Oliveira *et al*., 2011
LBP5046-26012	*Clupeacharax anchoveoides*	HQ171337	Oliveira *et al*., 2011
LBP473324984	*Compsura heterura*	HQ171332	Oliveira *et al*., 2011

LBP536-7140	*Copella nattereri*	HQ171431	Oliveira *et al*., 2011
LBP3267-20057	*Creagrutus peruanus*	HQ171279	Oliveira *et al*., 2011
MCP-28945	*Ctenobrycon hauxwellianus*	FJ749010	Javonillo *et al*., 2010
--	*Ctenobrycon hauxwellianus*	FJ944726	Vera *et al.,* 2009*
QCAZ2606	*Ctenobrycon hauxwellianus*	KT277930	Present Study
QCAZ2609	*Ctenobrycon hauxwellianus*	KT277931	Present Study
LBP3759-22034	*Curimatella dorsalis*	HQ171290	Oliveira *et al*., 2011
LBP1619-11672	*Cynodon gibbus*	HQ171241	Oliveira *et al*., 2011
QCAZ2585	*Cynopotamus amazonus*	KT277901	Present Study
LBP3225-19449	*Cynopotamus kincaidi*	HQ171271	Oliveira *et al*., 2011
LBP6132-29515	*Cynopotamus venezuelae*	HQ171359	Oliveira *et al*., 2011
LBP6827-33065	*Deuterodon iguape*	HQ171366	Oliveira *et al*., 2011
LBP7526-35371	*Distichodus* sp	HQ171385	Oliveira *et al*., 2011
LBP4038-22896	*Engraulisoma taeniatum*	HQ171299	Oliveira *et al*., 2011
LBP4038-22897	*Engraulisoma taeniatum*	HQ171300	Oliveira *et al*., 2011
MCP-28907	*Galeocharax knerii*	FJ749024	Javonillo *et al*., 2010
LBP4070-22975	*Gasteropelecus sternicla*	HQ171304	Oliveira *et al*., 2011
STRI AM208 19868	*Gasteropelecus maculatus*	GQ368213	Abe *et al.,* 2009*
QCAZ2855	*Gasteropelecus* sp1	KT277908	Present Study
LBP2753-18519	*Gephyrocharax atracaudatus*	HQ171265	Oliveira *et al*., 2011
AMNH233275	*Gephyrocharax* sp	AY788014	Calcagnotto *et al*., 2005
U34007	*Gephyrocharax* sp	U34007	Ortí y Meyer, 1997*
QCAZ2913	*Gephyrocharax sp*	KT277899	Present Study
LBP4507-24358	*Glandulocauda melanogenys*	HQ171320	Oliveira *et al*., 2011
LBP4495-24494	*Gnathocharax steindachneri*	HQ171325	Oliveira *et al*., 2011
RJ-DNA-17	*Gymnocorymbus ternetzi*	FJ749019	Javonillo *et al*., 2010

LBP3737-21989	*Gymnocorymbus ternetzi*	HQ171287	Oliveira *et al*., 2011
QCAZ2834	*Gymnocorymbus thayeri*	KT277888	Present Study
QCAZ2569	*Gymnocorymbus thayeri*	KT277887	Present Study
LBP5967-28455	*Hasemania* sp	HQ171354	Oliveira *et al*., 2011
LBP6847-33168	*Hemibrycon taeniurus*	HQ171369	Oliveira *et al*., 2011
AMNH233395	*Hemigrammus bleheri*	AY788017	Calcagnotto *et al*., 2005
AMNH233396	*Hemigrammus erythrozonus*	AY788023	Calcagnotto *et al*., 2005
LBP6292-29419	*Hemigrammus marginatus*	HQ171357	Oliveira *et al*., 2011
AMNH233397	*Hemigrammus rodwayi*	AY788034	Calcagnotto *et al*., 2005
LBP7604-36267	*Hemigrammus ulreyi*	HQ171394	Oliveira *et al*., 2011
QCAZ2604	*Hemigrammus unlineatus*	KT277893	Present Study
LBP1725-12849	*Hemiodus immaculatus*	HQ171246	Oliveira *et al*., 2011
LBP1221-25846	*Henochilus wheatlandii*	HQ171335	Oliveira *et al*., 2011
LBP7527-35373	*Hepsetus odoe*	HQ171387	Oliveira *et al*., 2011
LBP4494-24485	*Heterocharax macrolepis*	HQ171323	Oliveira *et al*., 2011
LBP487224954	*Heterocheirodon yatai*	HQ171330	Oliveira *et al*., 2011
LBP5539-27219	*Hoplias malabaricus*	HQ171346	Oliveira *et al*., 2011
LBP4495-24489	*Hoplocharax goethei*	HQ171324	Oliveira *et al*., 2011
RJ-DNA6	*Hyphessobrycon anisitsi*	FJ749004	Javonillo *et al*., 2010
QCAZ2634	*Hyphessobrycon copelandi*	KT277924	Present Study
AMNH233428	*Hyphessobrycon eques*	AY788022	Calcagnotto *et al*., 2005
RJ-DNA-10	*Hyphessobrycon erythrostigma*	FJ749015	Javonillo *et al*., 2010
RJ-DNA-4	*Hyphessobrycon herbertaxelrodi*	FJ749013	Javonillo *et al*., 2010
RJ-DNA16	*Hyphessobrycon megalopterus*	FJ749018	Javonillo *et al*., 2010
LBP7613-36932	*Hyphessobrycon megalopterus*	HQ171397	Oliveira *et al*., 2011
LBP1049-8939	*Hyphessobrycon reticulatus*	HQ171436	Oliveira *et al*., 2011

UFRGS-11129-EC325B	*Hyphessobrycon uruguayensis*	HM562764	Thomaz *et al*., 2010*
LBP4266-23840	*Iguanodectes geisleri*	HQ171316	Oliveira *et al*., 2011
AMNH233400	*Inpaichthys kerri*	AY788039	Calcagnotto *et al*., 2005
LBP7067-34380	*Jupiaba anteroides*	HQ171381	Oliveira *et al*., 2011
MCP-32548	*Jupiaba polylepis*	FJ749027	Javonillo *et al*., 2010
QCAZ2590	*Jupiaba* sp	KT277929	Present Study
QCAZ2942	*Knodus beta*	KT277898	Present Study
QCAZ2868	*Knodus breviceps*	KT277896	Present Study
QCAZ2962	*Knodus breviceps*	KT277897	Present Study
LBP756915818	*Knodus meridae*	HQ171257	Oliveira *et al*., 2011
RJ2009-MCP-40394	*Knodus* sp a	FJ749028	Javonillo *et al*., 2010
RJ2009-MCP-33083	*Knodus* sp t	FJ749029	Javonillo *et al*., 2010
AMNH233223	*Knodus* sp	AY788041	Calcagnotto *et al*., 2005
LBP5033-25982	*Kolpotocheirodon theloura*	HQ171336	Oliveira *et al*., 2011
LBP4459-24381	*Leporinus fasciatus*	HQ171321	Oliveira *et al*., 2011
QCAZ2710	*Leporinus friderici*	KT277914	Present Study
LBP2340-15941	*Leporinus lacustris*	EU181598	Santos *et al*., 2007*
LBP3808-21936	*Leporinus paranensis*	EU181635	Santos *et al*., 2007*
LBP4137-23661	*Leptagoniates steindachneri*	HQ171311	Oliveira *et al*., 2011
LBP8094-37519	*Lignobrycon myersi*	HQ171402	Oliveira *et al*., 2011
LBP1225-38090	*Lophiobrycon weitzmani*	HQ171411	Oliveira *et al*., 2011
LBP603929061	*Macropsobrycon uruguayanae*	HQ171355	Oliveira *et al*., 2011
LBP3383-21274	*Mimagoniates inequalis*	HQ171282	Oliveira *et al*., 2011
--	*Moenkhausia comma*	FJ944730	Vera *et al.,* 2009*
QCAZ2893	*Moenkhausia comma*	KT277889	Present Study
QCAZ2580	*Moenkhausia copei*	KT277880	Present Study

QCAZ2951	*Moenkhausia copei*	KT277881	Present Study
QCAZ2858	*Moenkhausia cotinho*	KT277935	Present Study
QCAZ2901	*Moenkhausia grandisquamis*	KT277891	Present Study
QCAZ2920	*Moenkhausia grandisquamis*	KT277892	Present Study
--	*Moenkhausia lepidura*	FJ944731	Vera *et al.,* 2009*
--	*Moenkhausia melogramma*	FJ944732	Vera *et al.,* 2009*
QCAZ2849	*Moenkhausia oligolepis*	KT277934	Present Study
QCAZ2784	*Moenkhausia oligolepis*	KT277933	Present Study
--	*Moenkhausia sanctaefilomenae*	AY523588	Hubert *et al.,* 2005*
AMNH233419	*Moenkhausia sanctaefilomenae*	AY788054	Calcagnotto *et al*., 2005
RJ-DNA-12	*Moenkhausia sanctaefilomenae*	FJ749016	Javonillo *et al*., 2010
LBP6101-28443	*Moenkhausia xinguensis*	HQ171353	Oliveira *et al*., 2011
QCAZ2908	*Moenkhausia* sp1	KT277886	Present Study
QCAZ2769	*Moenkhausia* sp2	KT277884	Present Study
QCAZ2905	*Moenkhausia* sp2	KT277885	Present Study
QCAZ2845	*Myleus duriventre*	KT277918	Present Study
LBP2184-15570	*Myloplus rubripinnis*	HQ171252	Oliveira *et al*., 2011
LBP6104-27476	*Nanocheirodon insignis*	HQ171349	Oliveira *et al*., 2011
RJ-DNA-22	*Nematobrycon palmeri*	FJ749021	Javonillo *et al*., 2010
LBP4052-22932	*Odontostilbe fugitive*	HQ171302	Oliveira *et al*., 2011
LBP8105-37555	*Orthospinus franciscensis*	HQ171404	Oliveira *et al*., 2011
LBP4472-24425	*Paracheirodon axelrodi*	HQ171322	Oliveira *et al*., 2011
LBP9208-43156	*Paragoniates alburnus*	HQ171426	Oliveira *et al*., 2011
QCAZ2564	*Paragoniates* sp	KT277894	Present Study
QCAZ2781	*Paragoniates* sp	KT277895	Present Study
LBP1135-5635	*Parodon nasus*	HQ171429	Oliveira *et al*., 2011

QCAZ2733	*Parodon pongoensis*	KT277915	Present Study
LBP6105-35623	*Phenagoniates macrolepis*	HQ171391	Oliveira *et al*., 2011
LBP558227299	*Phenacogaster calverti*	HQ171347	Oliveira *et al*., 2011
QCAZ2903	*Phenacogaster pectinatus*	KT277900	Present Study
LBP8514-38382	*Piabarchus analis*	HQ171412	Oliveira *et al*., 2011
LBP5109-26150	*Piabucus melanostomus*	HQ171338	Oliveira *et al*., 2011
QCAZ2644	*Piabucus melanostomus*	KT277905	Present Study
LBP2598-17243	*Planaltina britskii*	HQ171262	Oliveira *et al*., 2011
LBP3732-21986	*Poptella paraguayensis*	HQ171286	Oliveira *et al*., 2011
LBP3230-19465	*Prionobrama paraguayensis*	HQ171272	Oliveira *et al*., 2011
LBP-23663	*Prionobrama filigera*	JQ820086	Tagliacollo *et al*., 2012
QCAZ2924	*Prionobrama filigera*	KT277921	Present Study
AMNH233305	*Prochilodus nigricans*	AY788075	Calcagnotto *et al*., 2005
QCAZ2836	*Prochilodus nigricans*	KT277912	Present Study
LBP612729514	*Prochilodus reticulatus*	HQ171358	Oliveira *et al*., 2011
LBP2862-18570	*Pseudocorynopoma heterandria*	HQ171268	Oliveira *et al*., 2011
QCAZ2719	*Pygocentrus nattereri*	KT277916	Present Study
LBP7146-33170	*Rachoviscus crassiceps*	HQ171371	Oliveira *et al*., 2011
LBP2755-18529	*Roeboides guatemalensis*	HQ171267	Oliveira *et al*., 2011
--	*Roeboides salvadoris*	FJ439411	Ornelas-García *et al.,* 2008
LBP8157-38066	*Roestes ogilviei*	HQ171410	Oliveira *et al*., 2011
LBP850-9025	*Salminus brasiliensis*	HQ171437	Oliveira *et al*., 2011
LBP373122121	*Serrapinnus calliurus*	HQ171291	Oliveira *et al*., 2011
LBP897241813	*Serrapinnus piaba*	HQ171416	Oliveira *et al*., 2011
QCAZ2906	*Serrapinus* sp	KT277923	Present Study
--	*Serrasalmus rhombeus*	AF283939	Orti *et al*., 2007*

--	*Serrasalmus rhombeus*	AF283937	Orti *et al*., 2007*
QCAZ2770	*Serrasalmus cf rhombeus*	KT277917	Present Study
--	*Steindachnerina guentheri*	FJ944748	Vera *et al.,* 2009*
LBP5185-26336	*Steindachnerina insculpta*	HQ171339	Oliveira *et al*., 2011
QCAZ2852	*Steindachnerina* sp	KT277913	Present Study
LBP4078-22994	*Stethaprion erythrops*	HQ171305	Oliveira *et al*., 2011
--	*Stethaprion erytrops*	FJ944734	Vera *et al.,* 2009*
LBP3758-22029	*Tetragonopterus argenteus*	HQ171289	Oliveira *et al*., 2011
--	*Tetragonopterus argenteus*	FJ944735	Vera *et al.,* 2009*
QCAZ2618	*Tetragonopterus argenteus*	KT277903	Present Study
QCAZ2713	*Tetragonopterus argenteus*	KT277904	Present Study
LBP8268-37556	*Tetragonopterus chalceus*	HQ171405	Oliveira *et al*., 2011
RJ DNA-34	*Thoracocharax stellatus*	FJ748992	Javonillo *et al*., 2010
QCAZ2649	*Thoracocharax* sp	KT277906	Present Study
QCAZ2664	*Thoracocharax* sp	KT277907	Present Study
--	*Triportheus albus*	FJ944736	Vera *et al.,* 2009*
QCAZ2699	*Triportheus* cf *albus*	KT277909	Present Study
QCAZ2846	*Triportheus* cf *albus*	KT277910	Present Study
AMNH233403	*Triportheus angulatus*	AY788082	Calcagnotto *et al*., 2005
QCAZ2889	*Triportheus auritus*	KT277911	Present Study
--	*Triportheus magdalenae*	FJ944738	Vera *et al.,* 2009*
LBP39-3503	*Triportheus nematurus*	HQ171383	Oliveira *et al*., 2011
LBP2663-15580	*Triportheus orinocensis*	HQ171253	Oliveira *et al*., 2011
LBP-39-3501	*Triportheus pantanensis*	GQ368200	Abe *et al.,* 2009*
--	*Triportheus paranensis*	U33623	Ortí y Meyer, 1997*
LBP5145-33166	*Tyttocharax madeirae*	HQ171368	Oliveira *et al*., 2011

LBP3074-19694	*Xenagoniates bondi*	HQ171274	Oliveira *et al*., 2011
LBP9054-42218	*Xenurobrycon pteropus*	HQ171423	Oliveira *et al*., 2011

***References not cited in the article**

- Nakatani M, Miya M, Mabuchi K, Saitoh K, Nishida M (2011) Evolutionary history of Otophysi (Teleostei), a major clade of the modern freshwater fishes: Pangean origin and Mesozoic radiation. BMC Evol. Biol. 11: 177

- Tagliacollo VA, Souza-Lima R, Benine RC, Oliveira C (2012) Molecular phylogeny of Aphyocharacinae (Characiformes, Characidae) with morphological diagnoses for the subfamily and recognized genera. Mol. Phylogenet. Evol. In press.

- Thomaz AT, Malabarba LR, Bonatto SL (2010) The phylogenetic placement of *Hollandichthys* Eigenmann 1909 (Teleostei: Characidae) and related genera. Mol. Phylogenet. Evol. 57:1347-1352.

- Vera M, Fernandez C, Guisande C, Pelayo P (2009) Trophic and body shape characters that promote diversification in Neotropical Characiforms: Integration between phylogeny and community ecology. Unpublished. Genetics, University of Santiago de Compostela, Facultad de Veterinaria, Campus Lugo, Avda. Carballo Calero s/n, Lugo 27002, Spain

- Abe KT, Castro RMC, Benine RC, Foresti F, Oliveira C (2009) Molecular phylogeny of the Gasteropelecidae (Ostariophysi:Characiformes) and their hypothesized phylogenetic position among the Characiformes. Unpublished. Departamento de Morfologia, Universidade Estadual Paulista (Julio de Mesquita Filho), Distrito de Rubiao Junior s/n, Botucatu, Sao Paulo 18618-000, Brazil.

- Orti G (1997) The radiation of characiform fishes and the limits of resolution of mitocondrial ribosomal DNA sequences. Syst. Biol. 46:75-100.

- Santos GSA, Britski, HA, Foresti F, Oliveira C (2007) Comparative molecular analysis among species of *Leporinus* (Ostariophysi: Characiformes: Anostomidae) from Sao Francisco and La Plata Basins. Unpublished, direct submission. Morfologia, Instituto de Biociencias-UNESP, Rubiao Junior, Botucatu, Sao Paulo 18618000, Brazil.

- Hubert N, Bolillo C, Paugy D (2005) Does elision account for molecular saturation: case study based on mitochondrial ribosomal DNA among Characiform fishes (Teleostei: Ostariophysii). Mol. Phylogenet. Evol. 35: 300-308.
